# Association between FNDC5 Deficiency and Compromised Mitochondrial Integrity and Biogenesis in Kidney Disease

**DOI:** 10.7150/ijms.130226

**Published:** 2026-09-03

**Authors:** Chien-Wei Huang, Chia-Jung Li, Junne-Ming Sung, Yau-Sheng Tsai, Jin-Shuen Chen

**Affiliations:** 1 Institute of Clinical Medicine, College of Medicine, National Cheng Kung University, Tainan, Taiwan.; 2 Division of Nephrology, Department of Internal Medicine, Kaohsiung Veterans General Hospital, Kaohsiung, Taiwan.; 3 School of Medicine, College of Medicine, National Yang Ming Chiao Tung University, Taipei, Taiwan.; 4 Department of Obstetrics and Gynecology, Kaohsiung Veterans General Hospital, Kaohsiung 813, Taiwan.; 5 Institute of BioPharmaceutical Sciences, National Sun Yat-sen University, Kaohsiung 804, Taiwan.; 6 National Museum of Marine Biology & Aquarium, Pingtung 944, Taiwan.; 7 Division of Nephrology, Department of Internal Medicine, National Cheng Kung University Hospital, Tainan, Taiwan.; 8 Institute of Precision Medicine, National Sun Yat-Sen University, Kaohsiung, Taiwan.

**Keywords:** FNDC5, mitochondrial dynamics, kidney injury

## Abstract

**Background:**

Mitochondrial dysfunction is a hallmark of acute and chronic kidney diseases. This study investigates the expression landscape and potential association of FNDC5 with renal injury.

**Methods:**

We integrated human scRNA-seq, mouse spatial transcriptomics (ischemia-reperfusion injury model), *in vitro* high-glucose (HG) models in proximal tubule cells and podocytes, and a constitutive *Fndc5* knockout mouse model. Candidate transcription factor occupancy at the FNDC5 promoter was examined by ChIP-qPCR, and associations were assessed in a non-insulin-dependent diabetes mellitus mouse model.

**Results:**

scRNA-seq localized *FNDC5* to specific tubules, showing downregulation in diabetic nephropathy. Spatial transcriptomics confirmed a distinct loss of cortical *Fndc5* following ischemia-reperfusion injury. *In vitro*, HG treatment suppressed FNDC5, reduced cell viability, and triggered mitochondrial fragmentation, evidenced by increased DRP1 phosphorylation and downregulated MFN1/OPA1. ZEB1 and CREB1 were identified as candidate transcription factors showing occupancy at the FNDC5 promoter. Genetic deletion of *Fndc5* reproduced this fission-permissive shift and reduced PGC1α expression under basal, non-diabetic conditions, providing loss-of-function evidence that *Fndc5* deficiency is sufficient to reproduce this phenotype, independent of any additional metabolic stressor. *In vivo*, diabetic mice exhibited diminished FNDC5 levels, which correlated with an oxidative stress-associated marker (4-HNE) and reduced expression of the biogenesis-associated marker PGC1α.

**Conclusion:**

FNDC5 downregulation is a conserved feature of renal injury associated with mitochondrial fission, and genetic loss-of-function data indicates that Fndc5 deficiency is sufficient to reproduce selected features of this fission-permissive mitochondrial phenotype; whether FNDC5 acts through a defined downstream pathway remains to be established.

## Introduction

Diabetic kidney disease (DKD) remains the leading cause of chronic kidney disease (CKD) and kidney failure worldwide, and its burden continues to rise despite advances in glucose lowering therapy 1. Current clinical practice uses a multifactorial strategy that includes glycemic and blood pressure control, renin angiotensin system blockade, and kidney protective agents such as sodium glucose cotransporter 2 inhibitors (SGLT2i) and nonsteroidal mineralocorticoid receptor antagonists to slow DKD progression 2. Large outcome trials have shown that SGLT2i reduce major kidney endpoints in patients with CKD, including those with diabetes 3-5, and finerenone further lowers the risk of CKD progression and cardiovascular events in type 2 diabetes with CKD 6. However, many patients continue to develop progressive albuminuria, declining estimated glomerular filtration rate (eGFR), and tubulointerstitial fibrosis, highlighting the need to define residual mechanisms and identify additional therapeutic targets 2, 7.

DKD is increasingly recognized as a multicellular disease that involves coordinated injury across podocytes, endothelial cells, mesangial cells, and tubular epithelial cells, with maladaptive crosstalk promoting inflammation and fibrosis 8. Persistent metabolic stress drives oxidative damage and chronic inflammatory signaling, and metabolic memory can sustain cellular dysfunction even after improved metabolic control 9. Early structural changes, including disruption of the glomerular endothelial glycocalyx, may precede overt functional decline and contribute to albuminuria and microvascular dysfunction 10. These concepts support a shift from purely hemodynamic models toward integrated views that incorporate cellular stress adaptation, tissue remodeling, and immune activation as co determinants of progression.

A central mechanism in DKD pathogenesis is mitochondrial dysfunction. Renal cells, particularly proximal tubular epithelial cells, rely heavily on oxidative phosphorylation and are vulnerable to hyperglycemia induced ROS generation, impaired bioenergetics, and defective quality control. These abnormalities promote apoptosis, dysregulated autophagy or mitophagy, and inflammatory activation, ultimately driving fibrosis and nephron loss 11. Recent work highlights mitochondrial metabolic reprogramming in DKD, and mechanistic studies show that mitochondrial oxidative injury can rewire lipid metabolism in renal tubular epithelial cells, linking redox imbalance to downstream metabolic and structural deterioration 9, 12. Together, these findings position mitochondria as active drivers of DKD progression rather than passive bystanders. In addition, mitochondrial dynamics and biogenesis pathways are increasingly viewed as potential intervention points because they influence stress tolerance and recovery capacity in both tubular and glomerular compartments 13.

High resolution profiling approaches are refining the DKD map by resolving cell type and niche specific injury programs that are obscured in bulk analyses. Single cell and spatial transcriptomic studies have identified compartmentalized inflammatory and fibrotic trajectories and have shown that therapies remodel selective cellular programs, supporting the value of integrative omics for target discovery 14, 15. These datasets also provide a framework to link candidate genes to specific renal cell populations and to evaluate whether their regulation is conserved across species and disease stages. Within this context, the FNDC5 and irisin axis has emerged as a candidate link between systemic metabolism and renal cellular homeostasis. FNDC5 encodes the precursor of irisin, and accumulating evidence suggests that irisin related pathways modulate oxidative stress, inflammation, and mitochondrial function in multiple organs, including the kidney 16. In DKD, irisin has been reported to ameliorate renal injury in part by restoring autophagy in podocytes, implying a role in preserving glomerular epithelial integrity under diabetic stress 17. Inflammation also contributes to DKD progression, and macrophage and podocyte crosstalk has been proposed as an integrative node connecting immune activation, barrier dysfunction, and fibrosis 18. These observations raise the possibility that FNDC5 related signaling may intersect with mitochondrial quality control and immune associated stress pathways in a cell type dependent manner.

In the present study, we employed a multi-omics approach to decode FNDC5 regulation. While DKD is driven by chronic metabolic memory and sustained hyperglycemia, acute renal ischemia-reperfusion injury (IRI) provides a platform to observe the renal response to sudden, severe oxidative and energetic stress. By incorporating spatial transcriptomics of a mouse IRI model, we aimed to determine whether FNDC5 is also reduced during acute renal epithelial injury and to visualize its loss within the specific anatomical niches of the kidney cortex. Although DKD and IRI represent different clinical timelines, they share a common pathophysiological nexus centered on mitochondrial impairment and oxidative stress 19-21. Utilizing the acute IRI model for spatial transcriptomics allows for a high-resolution, topographical mapping of *Fndc5* expression loss during a synchronized and severe cellular insult, providing spatial insight into FNDC5 expression dynamics during acute renal oxidative stress 21, 22. We first leveraged scRNA-seq of human kidney samples and spatial transcriptomics of a mouse IRI model to construct a high-resolution map of FNDC5 expression and its topological loss during injury. Subsequently, we utilized in vitro models of high-glucose stress in human proximal tubule cells and mouse podocytes to determine how FNDC5 deficiency correlates with mitochondrial fragmentation and the expression of dynamics-related proteins (DRP1, MFN1, OPA1). Using bioinformatic analysis and ChIP-qPCR, we identified ZEB1 and CREB1 as candidate transcription factors exhibiting occupancy at the FNDC5 promoter. Finally, we validated these findings in vivo using a non-insulin-dependent diabetes mellitus (NIDDM) mouse model, examining the correlation between FNDC5 levels, oxidative stress, and mitochondrial biogenesis markers. Our findings provide insights into the expression and regulatory landscape of FNDC5 and are consistent with its potential involvement in mitochondrial dynamics, supporting it as a candidate pathway in renal injury that warrants further functional investigation.

## Materials and Methods

### Single-cell RNA sequencing (scRNA-seq) analysis

Publicly available scRNA-seq data from human kidney samples (comprising 3 Control and 3 Diabetes donors) were obtained from the Gene Expression Omnibus (GEO) database under accession number GSE131882 23. Raw data were processed to filter low-quality nuclei. Unsupervised clustering and visualization were performed using Seurat R package. Dimensionality reduction was achieved using t-Distributed Stochastic Neighbor Embedding (t-SNE). Cell types were annotated based on the expression of canonical markers, including LRP2 (PCT), UMOD (LOH), SLC12A3 (DCT), AQP2 (CD-PC), ATP6V1G3 (CD-IC), NPHS1 (PODO), PECAM1 (ENDO), and PTPRC (LEUK). The percentage of nuclei expressing FNDC5 was calculated and visualized for Control and Diabetes groups.

### Spatial transcriptomics

Spatial transcriptomics was performed on frozen kidney sections obtained from 129/SvEv mice. The study included a sham-operated control group (Sham) and an ischemia-reperfusion injury group harvested at 12 hours post-reperfusion (IRI 12h), with each section representing an independent biological replicate (Sham: 1,774 spots; IRI 12h: 1,780 spots) analyzed using the 10x Genomics Visium platform. Tissue sections were processed using the 10x Genomics Visium platform. Hematoxylin and Eosin (H&E) staining was performed for morphological reference. Spatial gene expression was analyzed for key renal markers (*Aqp2*, *Lrp2*, *Nphs1*) and *Fndc5*. Data were visualized as spatial feature plots displaying the mean UMI counts and the percentage of spots expressing the target genes. Because this dataset comprised a single tissue section (one biological specimen) per condition, spatial gene expression was analyzed descriptively (mean UMI counts and percentage of positive spots) and no inferential statistical testing was performed; these data are therefore exploratory.

### Animal models

Mice were housed in individually ventilated cages (IVC) within the positive pressure area of the Laboratory Animal Center at Kaohsiung Veterans General Hospital under controlled environmental conditions, including a temperature of 20-25°C, 50-60% humidity, and a 12-hour light/dark cycle, with *ad libitum* access to food and water (Protocol No. IACUC-2408-2607-23115-NHRI). For establishment of an NIDDM model, six-week-old wild-type (WT) mice were divided into two groups: a WT control group and an NIDDM group. WT control mice were fed a standard chow diet (1310, Altromin) for 10 weeks. In contrast, mice in the NIDDM group were fed a high-fat diet (D12492, Research Diets) for 10 weeks. To induce diabetes, seven-week-old mice in the NIDDM group were fasted for 12 hours and subsequently administered a single intraperitoneal injection of streptozotocin dissolved in 0.1 M citrate buffer (pH 4.5) at a dosage of 100 mg/kg body weight, with successful induction confirmed by fasting blood glucose levels exceeding 200 mg/dL measured one week post-injection. Body weight was recorded weekly to monitor changes in the mice. Blood and urine samples were collected and analyzed. Fasting blood glucose levels were monitored using the Accu-Chek Instant system (Roche). Serum concentration of creatinine was measured using the Ortho-Clinical Diagnostics VITROS 350 System. Urine samples were collected using metabolic cages (SN-783, Hinano). Urinary protein concentration was quantified using a bicinchoninic acid protein assay kit (Pierce), and proteinuria was calculated as the ratio of urinary protein to urinary creatinine (uPCR). Urine creatinine levels were measured using the Ortho-Clinical Diagnostics VITROS 350 System. When the mice were 15 weeks old, samples of kidneys were collected following mice euthanasia. Tissue samples were quickly frozen and stored at -80°C.

### Cell culture and treatments

HK2 human proximal tubule cells and conditionally immortalized mouse podocytes were selected as established, well-characterized models of the two major renal epithelial lineages implicated in diabetic kidney injury, rather than as a direct representation of the specific FNDC5-high subpopulations identified in the human snRNA-seq dataset. Podocyte FNDC5 transcript detection was comparatively sparse in the human dataset; the podocyte experiments were therefore designed to test whether FNDC5 protein and its associated mitochondrial phenotype are present and glucose-responsive in this cell type despite low baseline transcript detection, consistent with prior reports linking irisin to podocyte autophagy and injury. Human proximal tubular epithelial Cells (PTCs; HK2) and conditionally immortalized Mouse Podocytes were cultured in DMEM/F12 medium supplemented with 10 % fetal bovine serum. For glucose stimulation experiments, cells were incubated in Normal Glucose (NG, 5.5 mM) or High Glucose (HG, 30 mM) medium for 48-72 hours. To exclude the effect of osmotic pressure, mannitol (MAN) was used as an osmotic control (5.5 mM glucose + 24.5 mM mannitol, total 30 mM). Cell viability was assessed using CCK-8 assay.

### Mitochondrial morphology analysis

Cells were stained with MitoTracker Red to visualize mitochondrial structure. Fluorescence images were acquired using a confocal microscope. Mitochondrial morphology was quantified using MicroP software plugin in ImageJ. Images were preprocessed using adaptive thresholding to segment mitochondrial structures, and objects smaller than 10 pixels were excluded to remove background noise. The MicroP algorithm automatically classified mitochondrial morphologies into fragmented, intermediate, and tubular networks based on predefined morphological features. For visualization and network analysis, mitochondrial structures were further binarized and skeletonized to evaluate network characteristics. Quantitative parameters, including mitochondrial length and network complexity, were subsequently compared between groups. Images were acquired from randomly selected fields of view in each sample, and image analysis was performed using automated image processing pipelines to minimize observer bias. For each experimental group, images were acquired from 10 randomly selected fields of view, comprising approximately 100 cells per group, from at least three to five independent biological replicates.

### Western blot analysis

Western Blot Analysis Total protein was extracted from cells using RIPA buffer containing protease and phosphatase inhibitors and quantified via BCA assay. Equal amounts of protein were separated by SDS-PAGE and transferred onto PVDF or Nitrocellulose membranes. Following blocking with 5% non-fat milk for 1 hour at room temperature, membranes were incubated overnight at 4°C with primary antibodies against FNDC5 (Proteintech, 23995-1-AP), DRP1 (Abclonal, A2586), pDRP1 (Ser616) (Abcam, ab314755), FIS1 (Abclonal, A5821), MFN1 (Abclonal, A9880), OPA1 (Abclonal, A9833), GAPDH (Proteintech, 60004-1-IG), and β-actin (GeneTex, GTX109639). Membranes were then incubated with HRP-conjugated secondary antibodies, and immunoreactive bands were visualized using ECL substrate. Band intensities were quantified using ImageJ software. Protein levels were normalized to GAPDH or β-actin loading controls as indicated. The pDRP1 protein levels were normalized to total DRP1. Relative protein expression was calculated as fold change compared with the control group. The original blots were provided in Supplementary Fig. S1.

### ChIP-qPCR

Potential transcription factor binding sites within the *FNDC5* genomic locus (chr1:33,325,113-33,344,564, GRCh37/hg19 assembly) were identified using the UCSC Genome Browser utilizing ENCODE ChIP-seq tracks. For experimental validation, Chromatin Immunoprecipitation (ChIP) assays were performed in HK2 transfected with either an *Fndc5* overexpression vector (OE Fndc5) or an empty vector control (OE vehicle). Cross-linked chromatin was immunoprecipitated using primary antibodies against CEBPB (Proteintech, 23431-1-AP), ZEB1 (Proteintech, 21544-1-AP), and CREB1 (Proteintech, 12208-1-AP). The enrichment of DNA fragments corresponding to the *FNDC5* promoter region was quantified via qPCR, and the results were calculated as fold enrichment relative to the control. Prior to immunoprecipitation, 5% of the total chromatin was reserved as input and used to normalize ChIP-qPCR signals (% input method). This assay was performed under FNDC5-overexpression conditions and did not include a matched IgG isotype negative control or a validated positive-control locus; results are therefore reported as candidate occupancy/enrichment rather than confirmed endogenous transcription-factor binding.

### *Fndc5* knockout mice

*Fndc5* wild-type (WT) and knockout (KO) mice (total n = 28; six weeks of age; body weight 16-25 g; 15 females and 13 males) were used in this study. Founder mice carrying the Fndc5^em1Jsc^ allele (C57BL/6JNarl-Fndc5^em1Jsc^) were generated by CRISPR/Cas9-mediated targeting of Fndc5 exons 2 and 3 through The Jackson Laboratory's custom gene-targeting service, and were imported and backcrossed onto the C57BL/6JNarl background at the National Laboratory Animal Center (Taipei, Taiwan). This KO line was also used in our related study of *Fndc5* in diabetic nephropathy 24, where the generation and validation protocol is described in full. Tail tip tissue samples (approximately 1 mm) were collected from 4-week-old mice under isoflurane anesthesia (4-5% for induction, 1-3% for maintenance) for PCR-based genotyping to confirm the targeted alleles. Mice were housed and maintained as described above. Animal experiments involving *Fndc5* KO mice were approved by the Ethics Committee for Animal Care and Use at Kaohsiung Veterans General Hospital, Kaohsiung, Taiwan (Approval No. IACUC-2022-2023-A038).

### RNA extraction and quantitative PCR (RT-qPCR)

Total RNA was extracted from mouse kidney tissues using RNAzol reagent. cDNA was synthesized using TAKARA Reverse Transcription Kit. RT-qPCR was performed using SYBR Green on a Applied Biosystems 7000 Real-Time PCR System. The relative mRNA expression of Fndc5/FNDC5 was calculated using the 2⁻ΔΔCt method, with Gapdh/GAPDH as the reference gene and the WT control group used as the calibrator sample.

### Periodic acid-Schiff (PAS) staining

PAS staining was used to evaluate the condition of the glomerular basement membrane, mesangial matrix and kidney tubule. Paraffin-embedded kidney sections from WT and NIDDM mice were deparaffinized and rehydrated. The sections were stained using the ScyTek Periodic Acid Schiff (PAS) Stain Kit, and hematoxylin was then applied to counterstain the nuclei.

### Immunohistochemistry (IHC)

Paraffin-embedded kidney sections from WT and NIDDM mice were deparaffinized and rehydrated. Antigen retrieval was performed using Citrate buffer pH6.0. Sections were incubated with primary antibodies against FNDC5, 4-Hydroxynonenal (4-HNE, Bioss, bs-6313R), and peroxisome proliferator-activated receptor gamma coactivator 1-alpha (PGC1α, Abclonal, A12348). Staining was visualized using a DAB substrate kit, and nuclei were counterstained with hematoxylin.

### Transmission electron microscopy

Kidney tissues were fixed in 2.5% glutaraldehyde and embedded using the Low Viscosity Embedding Media Spurr kit (Sigma-Aldrich). Ultrathin sections were double-stained with uranyl acetate and lead citrate and examined using a transmission electron microscope (JEM 1400 PLUS; JEOL) at Kaohsiung Veterans General Hospital.

### Statistical analysis

Data are presented as the mean ± standard deviation (SD). The number of independent biological replicates (n = 3 to 6) for each experiment is specified in the corresponding figure legends. For in vitro cell culture experiments, each n represents an independent biological replicate. Before statistical comparison, the Shapiro-Wilk test was employed to assess the normality of the data, and the F-test was used to evaluate the equality of variances. For data following a normal distribution, statistical comparisons between two groups were performed using a two-tailed Student's t-test. In cases where the assumptions of normality or equal variance were violated, the Mann-Whitney U test or Welch's t-test was applied, respectively. All statistical analyses were conducted using GraphPad Prism 8 (GraphPad Software, San Diego, CA, USA). A P value < 0.05 was considered statistically significant, with significance levels indicated as *P < 0.05, **P < 0.01, and ***P < 0.001.

## Results

### Single-nucleus transcriptomic profiling of *FNDC5* expression in human diabetic kidney

To characterize the cellular landscape and *FNDC5* expression patterns in human diabetic nephropathy, we analyzed a publicly available scRNA-seq dataset (GSE131882). Unsupervised clustering of 23,980 nuclei revealed major kidney parenchymal and immune cell types, visualizing distinct populations including the proximal convoluted tubule (PCT), loop of Henle (LOH), distal convoluted tubule (DCT), collecting duct (CD) subtypes (principal cells [CD-PC] and intercalated cells [CD-ICA, CD-ICB]), podocytes (PODO), parietal epithelial cells (PEC), endothelial cells (ENDO), mesangial cells (MES), and leukocytes (LEUK) (Fig. 1A). We next evaluated the cell-type-specific expression of *FNDC5* in control (CTRL) and diabetic (DIABETES) human kidneys. Feature plots projected onto the t-SNE space indicated sparse *FNDC5* signals distributed across specific tubular clusters in both conditions (Fig. 1B). To quantify these patterns, we examined the percentage of nuclei expressing *FNDC5* across identified cell types (Fig. 1C). In the control group, *FNDC5* expression was most prominent in the CD-ICA and specific PCT clusters. Comparing the two conditions, the dot plot visualization indicated a reduction in the percentage of *FNDC5*-expressing nuclei within the CD-ICA cluster in the diabetes group relative to the control. While *FNDC5* was detectable in other tubular segments (e.g., PCT, CD-PC), the expression frequency in glomerular cells (PODO, MES) and immune cells (LEUK) remained negligible in both groups.

### Spatial transcriptomic landscape of *Fndc5* and renal injury markers in murine IRI model

To investigate the spatial downregulation of *Fndc5* in the context of tissue architecture, we performed spatial transcriptomics on kidney sections from 129/SvEv mice subjected to sham operation or 12 hours of ischemia-reperfusion injury. H&E staining visualized the tissue morphology, with a comparable number of feature spots analyzed in both groups (Sham: 1,774; IRI 12h: 1,780) (Fig. 2). We first validated the spatial resolution using canonical kidney markers. The collecting duct marker *Aqp2* was correctly localized to the medullary region in both conditions. Quantitative analysis showed that *Aqp2* expression remained relatively stable following injury, suggesting the preservation of medullary transcriptomic identity at this time point. In contrast, the proximal tubule marker *Lrp2*, which was robustly expressed throughout the cortex in the Sham group, exhibited a marked decline in the IRI 12h group. This reduction in *Lrp2* signal intensity and spatial coverage reflects the susceptibility of the cortical proximal tubules to ischemic injury. Regarding the gene of interest, *Fndc5* exhibited a low basal expression profile in the Sham group, distributed sparsely across the tissue section. Following 12 hours of IRI, *Fndc5* transcripts became virtually undetectable, indicating a rapid loss of expression in the injured kidney. The glomerular marker *Nphs1* showed negligible expression in both groups, confirming that the analyzed tissue sections were primarily representative of the renal cortex and medulla without significant capture of glomerular units. Consequently, these spatial data — derived from a single biological specimen per condition and analyzed descriptively without inferential statistics — were used specifically to visualize the topographical loss of Fndc5 within the tubular compartments (Lrp2-positive proximal tubules) rather than the glomerulus, and should be interpreted as exploratory.

### Baseline mitochondrial characteristics and high glucose-induced mitochondrial fragmentation in kidney cells

To establish the baseline mitochondrial differences between tubular and glomerular compartments, we compared human proximal tubule cells (PTCs) and mouse podocytes under normal physiological conditions. Fluorescence microscopy revealed that human PTCs possess a dense, highly interconnected, and filamentous mitochondrial network, whereas mouse podocytes exhibit a relatively less dense mitochondrial architecture (Fig. 3A). Quantitative analysis of mitochondrial morphology confirmed that the median mitochondrial length was greater in PTCs compared to podocytes (Fig. 3B). Furthermore, the mitochondrial DNA to nuclear DNA ratio (mtDNA/nDNA), a proxy for mitochondrial mass and copy number, was markedly higher in PTCs than in podocytes (Fig. 3C), reflecting the high bioenergetic demand of the proximal tubule.

We next investigated the impact of hyperglycemic stress on these cell types. Exposure to HG conditions significantly reduced cell viability in both human PTCs (Fig. 4A) and mouse podocytes (Fig. 4B) compared to the NG controls. To exclude the effect of osmotic pressure, MAN was used as an osmotic control, which showed no significant impact on podocyte viability (Supplementary Fig. S2). To determine if this cytotoxicity was associated with mitochondrial structural remodeling, we visualized mitochondria using MitoTracker staining. In the NG group, both PTCs and podocytes maintained long, linear, and branched mitochondrial networks (Fig. 4C, D, left panels). In contrast, HG treatment induced severe mitochondrial fragmentation, characterized by the loss of tubular networks and the accumulation of punctate, swollen structures (Fig. 4C, D, right panels). Skeletonization and morphological classification further highlighted this transition. Under HG conditions, the mitochondrial population shifted from predominantly "linear" and "branched" phenotypes (green and purple annotations) to "globules" and "swollen" forms (blue and light blue annotations) in both cell types (Fig. 4E, F). Quantitative analysis corroborated these visual observations, showing a significant decrease in mitochondrial length in both human PTCs (Fig. 4G) and mouse podocytes (Fig. 4H) following HG exposure. Collectively, these data indicate that high glucose compromises cell viability and triggers extensive mitochondrial fragmentation in both tubular and glomerular cells.

### High glucose suppresses FNDC5 expression and disrupts mitochondrial dynamics-related proteins

To elucidate the molecular mechanism underlying the observed mitochondrial fragmentation, we evaluated the protein levels of FNDC5 and key regulators of mitochondrial dynamics (fusion and fission machinery) using Western blot analysis. In human PTCs, exposure to HG conditions resulted in a significant downregulation of FNDC5 protein levels compared to the NG control (Fig. 5A, C). Concurrently, the balance of mitochondrial dynamics proteins was shifted. The ratio of phosphorylated DRP1 (pDRP1-s616) to total DRP1, an indicator of mitochondrial fission activation, was significantly increased in the HG group. Interestingly, the expression of FIS1, another fission mediator, was decreased under HG conditions in PTCs. Regarding fusion machinery, both the outer membrane GTPase MFN1 and the inner membrane GTPase OPA1 were significantly downregulated in response to HG (Fig. 5C), suggesting a compromised fusion capacity. A similar pattern of dysregulation was observed in mouse podocytes. HG treatment led to a significant reduction in FNDC5 expression (Fig. 5B, D). Consistent with the findings in PTCs, podocytes exhibited an elevated pDRP1/DRP1 ratio and a significant decrease in the fusion proteins MFN1 and OPA1 (Fig. 5D). However, unlike in PTCs, the expression of FIS1 was significantly upregulated in podocytes under HG stress. Collectively, these data indicate that high glucose creates an imbalance in mitochondrial dynamics favoring fission over fusion, driven by increased DRP1 phosphorylation and the suppression of MFN1 and OPA1, alongside a consistent loss of FNDC5. In a separate rescue experiment, overexpression of FNDC5 (OE FNDC5) significantly attenuated the HG-induced downregulation of MFN2 — an additional, distinct outer-mitochondrial-membrane fusion protein not otherwise assessed in Fig. 5 — as well as the HG-induced alterations in FIS1 expression, suggesting a protective role of FNDC5 in maintaining mitochondrial fusion/fission protein balance under metabolic stress (Supplementary Fig. S3). We assessed intracellular reactive oxygen species (ROS) levels using the DCFDA fluorescence assay as an additional indicator related to mitochondrial stress. HG treatment was associated with significantly increased ROS production in renal cells compared with NG treatment. In contrast, the addition of recombinant Irisin (200 ng/mL) was associated with a reduction in HG-induced ROS accumulation (Supplementary Fig. S4). Given that elevated ROS levels are commonly linked to mitochondrial stress and impaired mitochondrial homeostasis, these findings provide supportive functional evidence consistent with a potential association between FNDC5/Irisin signaling and the preservation of mitochondrial integrity, beyond morphological observations alone.

### Identification of potential transcription factors occupying the FNDC5 promoter

To identify potential upstream transcriptional regulators of *FNDC5*, we utilized the UCSC Genome Browser to analyze chromatin immunoprecipitation sequencing (ChIP-seq) data from the ENCODE project (GRCh37/hg19 assembly). Examination of the *FNDC5* genomic locus (chr1:33,325,113-33,344,564) revealed distinct binding signals for several transcription factors, including ZEB1, CEBPB, and CREB1 (Fig. 6A). These signals coincided with genomic segments annotated by ChromHMM as active promoters (red) or enhancers (orange/yellow), suggesting a regulatory potential of these factors in *FNDC5* transcription. We further assessed the occupancy of selected candidates (CEBPB, ZEB1, and CREB1) to the *FNDC5* promoter region using ChIP-qPCR assays (Fig. 6B). Quantitative analysis demonstrated a statistically significant enrichment of ZEB1 (P = 0.0145) and CREB1 (P = 0.0401) at the *FNDC5* locus in the Fndc5-overexpressing (OE Fndc5) group compared to the vehicle control. Although CEBPB showed a trend toward increased enrichment, the difference did not reach statistical significance (P = 0.0713). These data indicate candidate occupancy/enrichment of ZEB1 and CREB1 at the FNDC5 regulatory region under the FNDC5-overexpression conditions used in this assay. Because the assay lacked a matched IgG isotype negative control and a validated positive-control locus, these findings should be interpreted as exploratory evidence of candidate occupancy rather than confirmed physical binding or endogenous transcriptional regulation. Coupled with ENCODE bioinformatic evidence, they identify ZEB1 and CREB1 as candidate upstream regulators of FNDC5 that warrant further validation — including assays performed under endogenous (non-overexpression) conditions with appropriate IgG and positive-control loci, and functional silencing/overexpression of ZEB1 and CREB1 — to establish their necessity in the stress-induced suppression of FNDC5.

### Downregulation of FNDC5 correlates with oxidative stress and reduced PGC1α expression in diabetic mice

To establish the diabetic nephropathy phenotype, we characterized the metabolic and renal profiles of the NIDDM mouse model. One week following streptozotocin injection, the NIDDM group exhibited significantly elevated fasting blood glucose levels compared to WT controls (NIDDM: >400 mg/dL vs. WT: ~120 mg/dL; P < 0.001), a trend that persisted throughout the study period. While body weight and serum creatinine levels showed no significant differences between the two groups at the time of sacrifice, the NIDDM mice developed progressive albuminuria, as evidenced by a significantly higher uPCR starting from the tenth week (Supplementary Fig. S5). These physiological changes, combined with the observed glomerular basement membrane thickening and mesangial expansion, confirm the successful induction of DKD in our model. We used PAS staining to assess the condition of kidney glomeruli (Supplementary Fig. S5). Compared with WT mice, NIDDM mice exhibited significant glomerular basement membrane thickening and notable mesangial expansion, with no marked differences observed in the renal tubules. Quantitative PCR analysis revealed a significant reduction in *FNDC5* mRNA levels in the NIDDM group compared to the WT control mice (Fig. 7A). The IHC staining further confirmed reduced FNDC5 expression in the kidneys of NIDDM mice compared with WT controls (Fig. 7B). To evaluate oxidative stress status, kidney sections were stained for 4-HNE, a lipid peroxidation marker. Compared with controls, NIDDM kidneys displayed increased 4-HNE staining intensity (Fig. 7C), consistent with enhanced oxidative stress in diabetic renal tissue. Furthermore, the expression of PGC1α, a master regulator of mitochondrial biogenesis and a known interactor of FNDC5, was substantially attenuated in the NIDDM group (Fig. 7D). These in vivo results suggest that the suppression of FNDC5 in the diabetic kidney is accompanied by an oxidative stress-associated readout (increased 4-HNE staining) and reduced expression of the biogenesis-associated marker PGC1α; direct measures of mitochondrial function were not assessed in this study.

### Genetic deletion of *Fndc5* reproduces mitochondrial dynamics imbalance and reduced PGC1α expression under basal conditions

To directly test whether loss of FNDC5 is sufficient to reproduce the mitochondrial phenotype observed under metabolic stress, we examined kidneys from *Fndc5* knockout (KO) mice under basal, non-diabetic conditions. Transmission electron microscopy revealed a shift in mitochondrial ultrastructure in *Fndc5* KO kidneys compared with WT controls, with mitochondria in the KO group appearing more rounded and less elongated than the filamentous networks observed in WT (Fig. 8A). Consistent with this structural change, quantitative RT-PCR showed a significant increase in *Drp1* mRNA expression (Fig. 8B) and a significant decrease in *Mfn1* mRNA expression (Fig. 8C) in *Fndc5* KO kidneys, indicating a basal shift toward a fission-permissive state in the absence of any additional metabolic stressor. Immunohistochemical staining confirmed efficient depletion of FNDC5 protein in the KO kidneys (Fig. 8D, E) and revealed a concomitant significant reduction in PGC1α-positive staining area (Fig. 8D, F), mirroring the reduced PGC1α expression observed in the NIDDM model (Fig. 7D). Together, these findings demonstrate that genetic deletion of *Fndc5* alone, independent of hyperglycemic or ischemic stress, is sufficient to shift mitochondrial dynamics-related markers toward a fission-permissive profile and reduce PGC1α expression, providing genetic loss-of-function evidence that complements the FNDC5 overexpression (gain-of-function) data described above.

## Discussion

In this study, we integrate human single cell evidence, murine spatial transcriptomics, mitochondrial phenotyping, and in vitro and *in vivo* validation to characterize the association of FNDC5 in diabetic kidney injury. Across datasets and models, FNDC5 signals were reduced in disease contexts and aligned with mitochondrial structural disruption, oxidative stress, and attenuation of mitochondrial biogenesis related markers. These observations are consistent with a model in which FNDC5 downregulation accompanies stress responsive programs associated with mitochondrial homeostasis in diabetic kidney disease.

A key contribution is the use of high-resolution transcriptomic frameworks to localize FNDC5 changes to specific renal compartments and cellular contexts. DKD is increasingly understood as a multicellular disease involving coordinated injury across glomerular and tubular units, together with immune and stromal responses that evolve with spatial heterogeneity. Single cell and spatial approaches have refined this view by resolving niche restricted injury states and treatment responsive programs that are not apparent in bulk tissue analyses 9, 12, 14, 15, 23. Within this conceptual framework, our findings suggest that FNDC5 regulation is not a uniform whole kidney event but rather part of compartment specific stress adaptation, which may help explain divergent results reported by studies using different sampling strategies, disease stages, and readouts. In this setting, our spatial and single cell observations suggest that FNDC5 regulation is not simply a uniform whole kidney event but reflects cell type specific and microenvironment dependent stress adaptation. The observation that FNDC5 is reduced in both the acute IRI model and chronic DKD models suggests that FNDC5 downregulation may occur under distinct forms of renal stress. Mitochondrial fragmentation, driven by unbalanced fission/fusion signaling, acts as a shared executioner of cell death in both ischemic and metabolic injuries 25, 26. We therefore regard the FNDC5-mitochondria relationship as a candidate, stress-responsive association rather than a demonstrated universal or fundamental regulatory axis, and we have removed language implying the latter 27, 28.

Our data consistently associate FNDC5 downregulation with altered markers of mitochondrial integrity. Mitochondrial dysfunction is now widely recognized as a contributor to DKD progression through impaired bioenergetics, excess ROS, defective quality control, and maladaptive signaling that promotes inflammation and fibrosis 25, 29. Consistent with this paradigm, we observed mitochondrial network remodeling and reduced mtDNA content in renal cell models under diabetic stress. These structural readouts are biologically meaningful because mitochondrial fragmentation can potentiate oxidative injury and lower stress tolerance in both tubular epithelial cells and podocytes 30, 31. Recent studies further underscore that diabetic kidneys undergo metabolic rewiring linked to mitochondrial oxidative injury, including lipid metabolic reprogramming in tubular cells 9, 12, 32.

Prior studies have established mitochondrial dynamics as a therapeutically actionable axis in DKD, with DRP1-driven fission signaling serving as a central determinant of mitochondrial fragmentation and cellular susceptibility to injury 25. Our findings suggest that FNDC5 downregulation accompanies this fission permissive state, and they motivate future functional tests to determine whether restoring FNDC5 can rebalance fission and fusion and improve mitochondrial resilience. The* in vivo* diabetic model strengthens the biological coherence of the pathway. In NIDDM kidneys, reduced FNDC5 staining was accompanied by increased 4-HNE signal and reduced PGC1α related immunoreactivity (Fig. 7). This pattern aligns with a diabetic tissue environment characterized by lipid peroxidation and weakened mitochondrial biogenesis programs, processes repeatedly implicated in progressive nephron dysfunction 12, 29, 33. Together with the *in vitro* findings, these results support a framework in which FNDC5 suppression is associated with oxidative stress-associated readouts and reduced expression of biogenesis-associated markers in diabetic kidneys. As noted above, we did not directly measure mitochondrial function (oxygen consumption, membrane potential, ATP production, mitophagy flux), and the present data should not be read as demonstrating definitive functional impairment or a biogenesis defect.

Our results also complement growing interest in the FNDC5 and irisin axis as an organ protective pathway. Irisin related biology has been linked to oxidative stress control, autophagy, and mitochondrial function across tissues, and experimental DKD studies report that irisin can ameliorate diabetic kidney injury in part by restoring autophagy in podocytes 17. Inflammation and immune parenchymal interactions also shape DKD outcomes, and macrophage podocyte crosstalk has been proposed as an integrative node connecting immune activation to barrier dysfunction and fibrosis 18, 34. A testable implication is that FNDC5 related mitochondrial stabilization could indirectly modulate inflammatory amplification, providing a mechanistic rationale for integrating mitochondrial and immune phenotyping in future FNDC5 focused studies.

Several limitations warrant consideration. First, our data establish consistent associations between FNDC5 suppression and mitochondrial injury markers under conditions of metabolic or ischemic stress, but they do not, by themselves, prove causality. To address this, we generated a constitutive *Fndc5* KO mouse model and found that genetic loss of *Fndc5* alone, under basal, non-diabetic conditions, was sufficient to shift Drp1/Mfn1 expression and reduce PGC1α (Fig. 8). These loss-of-function data indicate that *Fndc5* deficiency is sufficient to reproduce selected features of a fission-permissive mitochondrial state; they do not establish that FNDC5 acts through DRP1 and do not demonstrate a linear FNDC5-DRP1-mitochondrial injury pathway. However, this causal relationship remains to be established under disease-relevant hyperglycemic or ischemic stress conditions, and further genetic manipulations in stressed models are required to confirm it. Second, mitochondrial morphology and mtDNA content are informative but incomplete proxies for function, and future work should incorporate respiration, substrate utilization, and mitophagy flux measurements. The ROS and PGC1α readouts likewise provide supportive but incomplete evidence regarding mitochondrial integrity and biogenesis and should not be overinterpreted as mechanistic proof. Third, although FNDC5 overexpression partially preserved mitochondrial fusion-related proteins under high-glucose stress, and genetic deletion of *Fndc5* reproduced a shift toward increased *Drp1* and decreased *Mfn1* expression together with reduced PGC1α under basal conditions (Fig. 8), we did not test whether DRP1-related fission signaling is functionally required downstream of FNDC5 loss—for example, via DRP1 inhibition or silencing in the *Fndc5*-deficient background; the present data therefore do not establish a complete mechanistic pathway, and the model in Figure 9 should be regarded as a working hypothesis to be tested in future functional studies. Fourth, while our ChIP-qPCR and bioinformatic analyses identify occupancy of ZEB1 and CREB1 at the *FNDC5* promoter, these findings demonstrate promoter occupancy rather than regulatory directionality. Specifically, the current study does not include promoter-reporter (luciferase) assays or site-directed mutagenesis to confirm the functional significance of the predicted binding sites. Furthermore, the "conceptually inverted" design of performing ChIP under *FNDC5* overexpression conditions primarily validates site occupancy; future experiments involving the direct silencing or overexpression of ZEB1 and CREB1 will be necessary to fully decipher the upstream regulatory network. Fifth, the spatial transcriptomic findings from the acute IRI model should be interpreted as supportive evidence that FNDC5 can be reduced during acute tubular injury, and future studies will be required to determine whether these observations reflect shared or disease-specific mechanisms. Sixth, our ChIP-qPCR experiments were normalized to input chromatin (5% input) but did not include a matched IgG isotype negative control or an established positive-control locus; in the absence of these controls, the specificity of the observed ZEB1 and CREB1 enrichment cannot be fully distinguished from nonspecific chromatin recovery. Additionally, although mitochondrial morphology classification was performed using an automated image-processing pipeline (MicroP) to minimize subjective bias, field selection and image quantification were not performed by an observer blinded to group allocation. Future studies should incorporate IgG controls, a validated positive-control locus, and blinded image analysis to address these limitations. Finally, the relationship between local renal FNDC5 programs and circulating irisin remains complex and will require matched systemic measurements and renal cell type restricted manipulations.

## Conclusion

In conclusion, this study identifies FNDC5 downregulation as a feature associated with altered mitochondrial morphology and dynamics-related markers in the kidney. These findings suggest that FNDC5 is a candidate component of the cellular response to metabolic and ischemic stress in the kidney; whether it functions within a defined regulatory pathway, rather than as an associated marker, remains to be functionally established (Figure 9).

## Supplementary Material

Supplementary figures.

## Figures and Tables

**Figure 1:**
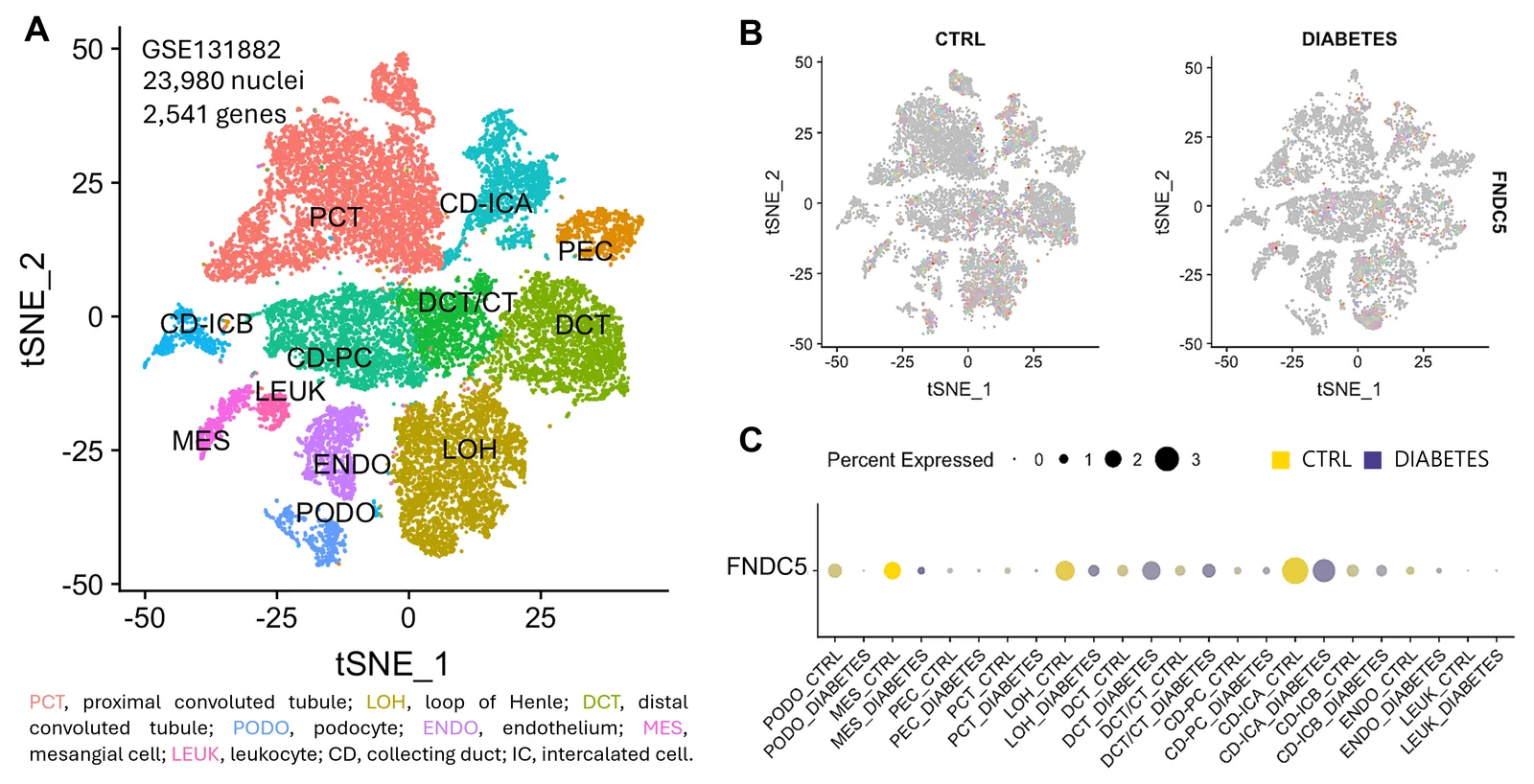
** scRNA sequencing analysis of *FNDC5* expression in human kidney tissue.** (A) t-SNE visualization of 23,980 nuclei from human kidney samples (GSE131882). Clusters are annotated by cell type: PCT, proximal convoluted tubule; LOH, loop of Henle; DCT, distal convoluted tubule; CT, connecting tubule; CD, collecting duct; PC, principal cell; IC, intercalated cell (subtypes A and B); PODO, podocyte; PEC, parietal epithelial cell; ENDO, endothelium; MES, mesangial cell; LEUK, leukocyte. (B) Feature plots showing the distribution of *FNDC5*-expressing nuclei in Control (CTRL) and Diabetes groups. (C) Dot plot comparing the percentage of cells expressing *FNDC5* across cell types between CTRL (yellow) and DIABETES (purple) groups. Dot size represents the percent of nuclei expressing the gene within each cluster.

**Figure 2:**
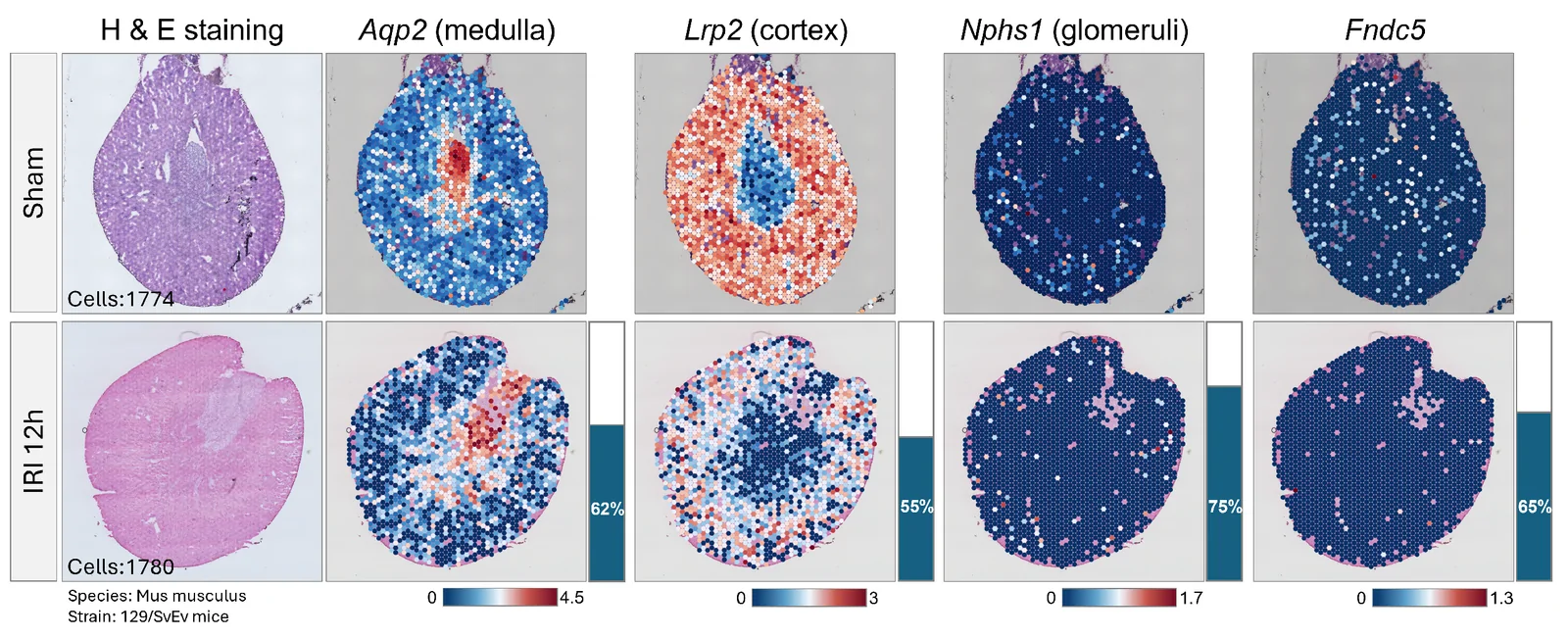
** Spatial transcriptomics analysis of *Fndc5* and renal markers in a mouse IRI model.** Spatial gene expression maps of kidney sections from Sham-operated and ischemia-reperfusion injury (IRI, 12 hours) mice (*Mus musculus*, strain 129/SvEv). Left panels: H&E staining of the corresponding tissue sections. The total number of feature spots (Cells) analyzed is indicated for each group (Sham: 1,774; IRI 12h: 1,780). Right panels: Spatial feature plots showing the expression distribution of *Aqp2* (medullary marker), *Lrp2* (cortical/proximal tubule marker), *Nphs1* (glomerular marker), and *Fndc5*. The spots are color-coded based on expression levels (Blue: low; Red: high).

**Figure 3:**
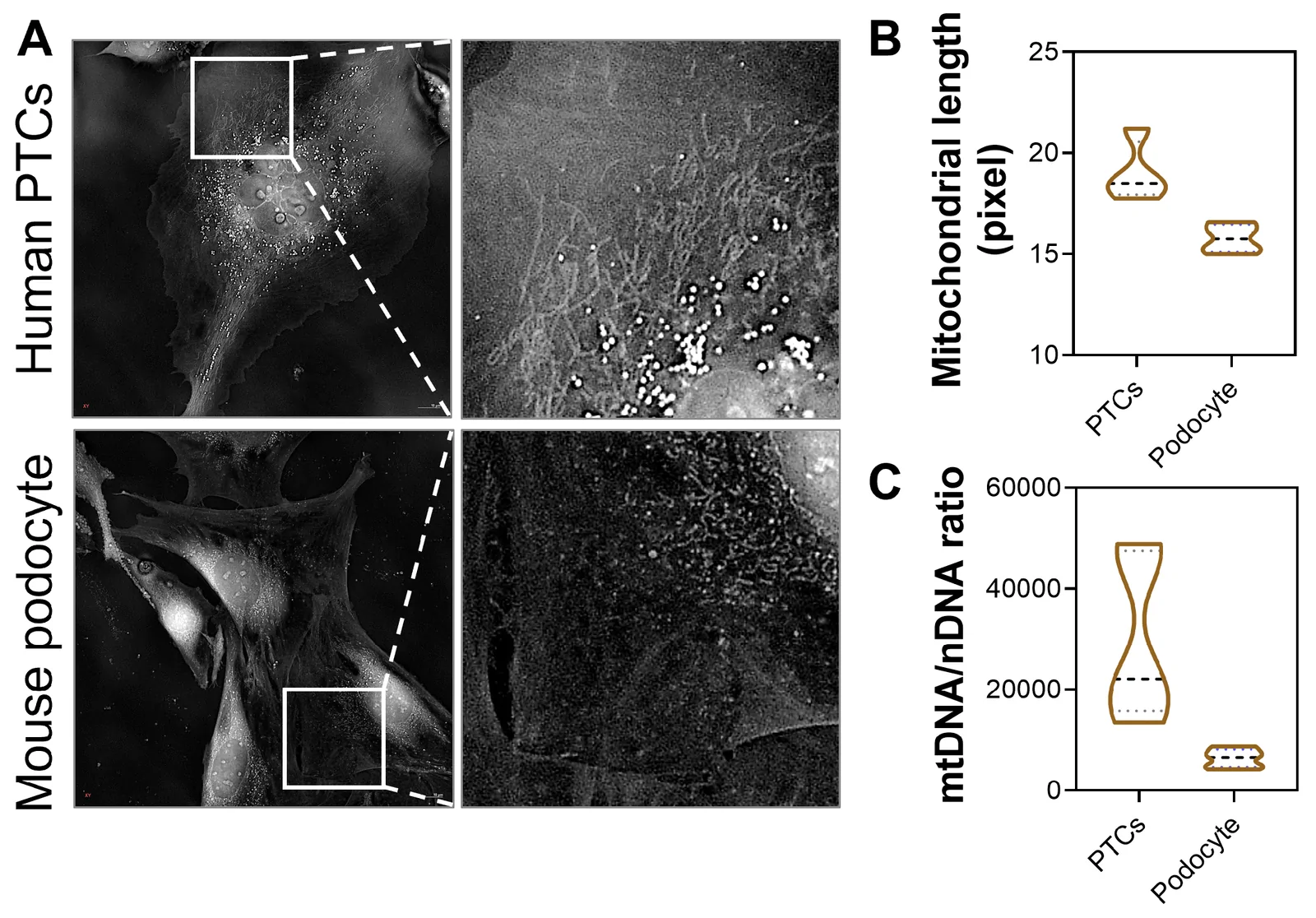
** Comparison of mitochondrial morphology and content between human PTCs and mouse podocytes.** (A) Representative fluorescence images showing the mitochondrial network in Human PTCs and Mouse podocytes. Boxed areas indicate regions of magnification shown in the right panels. (B) Violin plot quantifying mitochondrial length (in pixels) in PTCs and podocytes. (C) Violin plot comparing the mtDNA/nDNA ratio between PTCs and podocytes. The dashed lines within the violin plots represent the median and quartiles.

**Figure 4:**
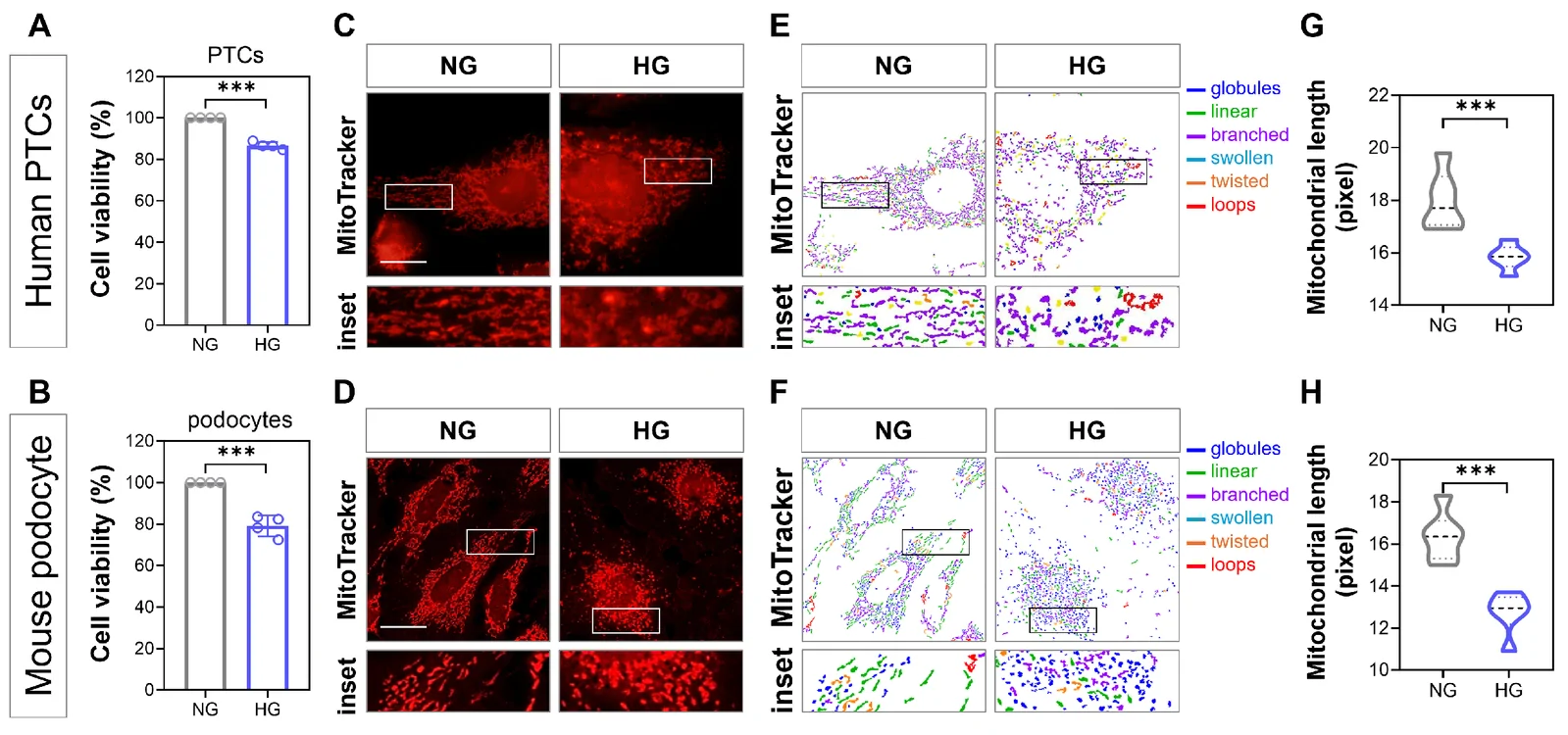
** High glucose induces cytotoxicity and mitochondrial fragmentation in kidney cells.** (A-B) Cell viability analysis of Human PTCs (A) and Mouse podocytes (B) cultured under Normal Glucose (NG) or High Glucose (HG) conditions. (C-D) Representative MitoTracker Red fluorescence images of Human PTCs (C) and Mouse podocytes (D) under NG and HG conditions. Scale bars = 20 μm. (E-F) Skeletonized images corresponding to the MitoTracker staining in panels C and D. Mitochondria are color-coded based on morphological classification: globules (blue), linear (green), branched (purple), swollen (light blue), twisted (orange), and loops (red). Note the shift towards globular and swollen phenotypes in the HG group. (G-H) Violin plots quantifying mitochondrial length (in pixels) in Human PTCs (G) and Mouse podocytes (H). *** P < 0.001.

**Figure 5:**
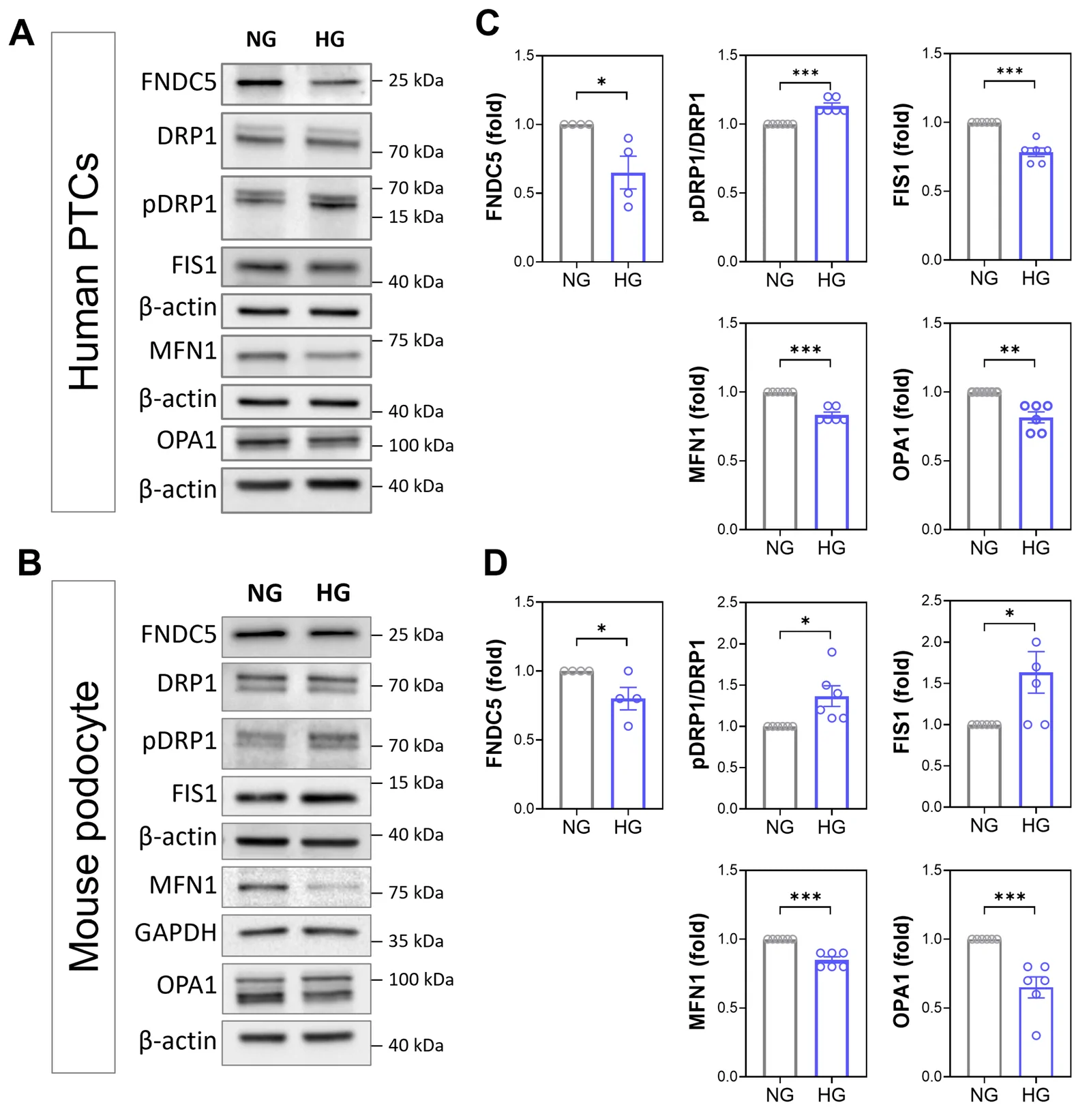
** Effect of high glucose on FNDC5 expression and mitochondrial dynamics proteins in human PTCs and mouse podocytes.** (A-B) Representative Western blots showing the protein levels of FNDC5, fission markers (DRP1, pDRP1, FIS1), and fusion markers (MFN1, OPA1) in Human PTCs (A) and Mouse podocytes (B) cultured under Normal Glucose (NG) or High Glucose (HG) conditions. pDRP1 was normalized to total DRP1 to assess its phosphorylation status. For all other proteins, normalization was performed against the corresponding loading control shown in the blots: in human PTCs, β-actin was used for all targets; in mouse podocytes, GAPDH was used for MFN1, while β-actin was used for FNDC5, FIS1, and OPA1. (C) Densitometric quantification of the indicated proteins in Human PTCs. (D) Densitometric quantification of the indicated proteins in Mouse podocytes. Data are expressed as fold change relative to the NG control. The pDRP1 levels were normalized to total DRP1 (pDRP1/DRP1 ratio). * P < 0.05, ** P < 0.01, and *** P < 0.001.

**Figure 6:**
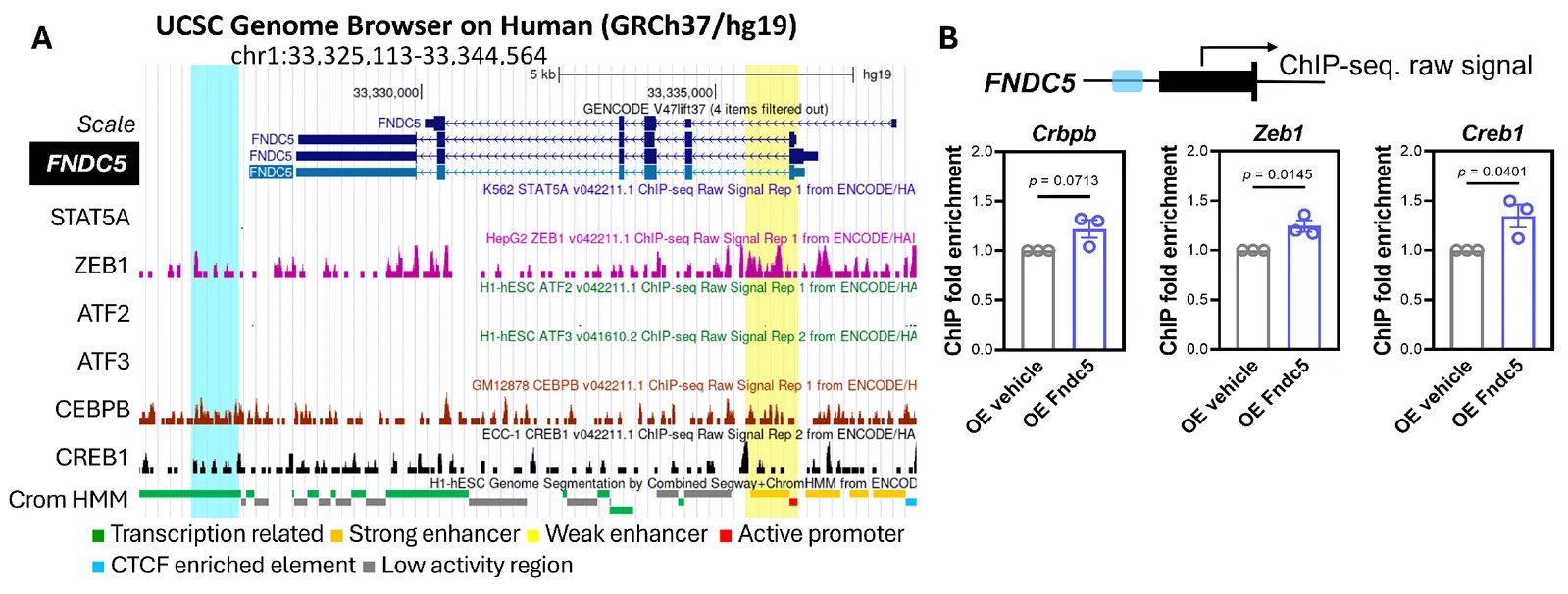
** Identification of candidate transcription factors occupying the FNDC5 locus.** (A) Visualization of the *FNDC5* gene locus on the UCSC Genome Browser (Human GRCh37/hg19 assembly). Tracks display ChIP-seq raw signals for potential transcription factors (STAT5A, ZEB1, ATF2, ATF3, CEBPB, CREB1) and ChromHMM chromatin state segmentation (Red: Active promoter; Orange/Yellow: Strong/Weak enhancer; Green: Transcription related; Blue: CTCF enriched element). (B) ChIP-qPCR analysis of candidate transcription-factor occupancy/enrichment. Quantification of ChIP fold enrichment for CEBPB, ZEB1, and CREB1 in HK2 cells transfected with vehicle (OE vehicle) or an Fndc5 overexpression construct (OE Fndc5).

**Figure 7:**
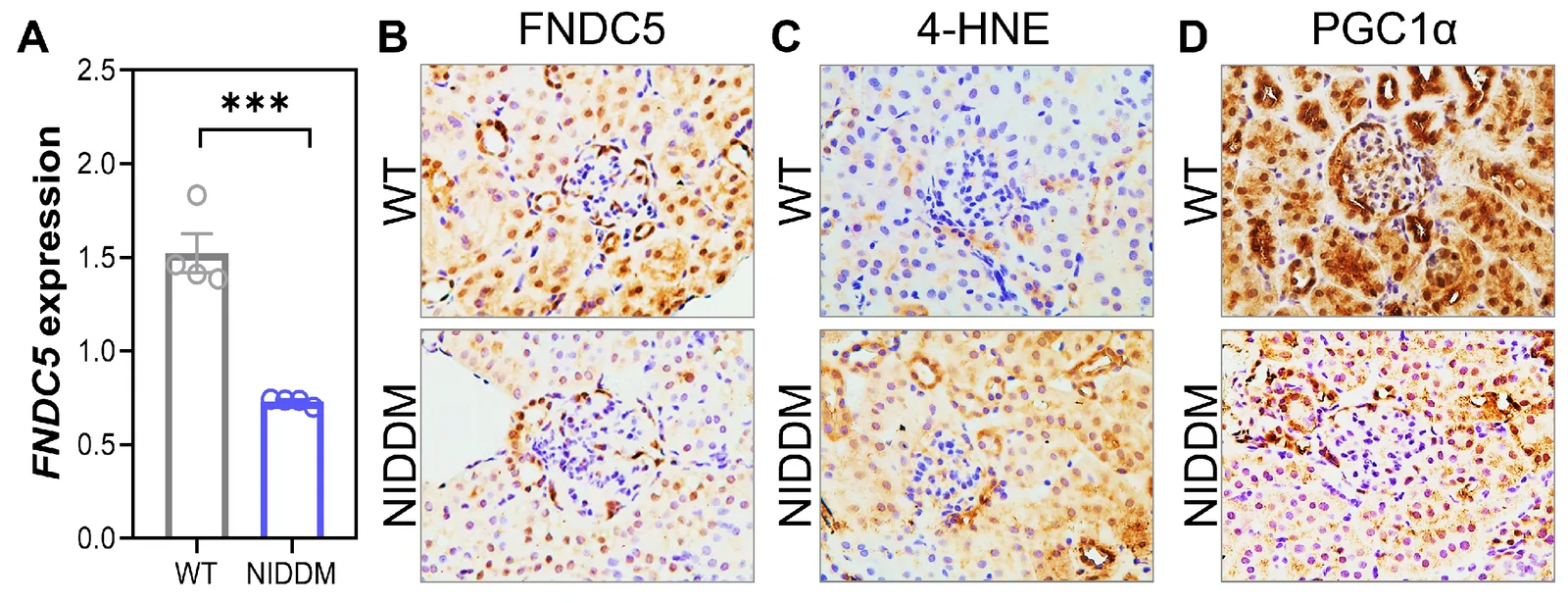
** Evaluation of FNDC5 expression, oxidative stress, and PGC1α levels in a diabetic mouse model.** (A) Quantitative RT-PCR analysis of *Fndc5* mRNA expression in kidney tissues from WT control and NIDDM mice. (B-D) Representative immunohistochemical staining of kidney sections from WT control and NIDDM mice. (B) Staining for FNDC5 protein, showing reduced expression in the NIDDM group. (C) Staining for 4-HNE, indicating increased accumulation of lipid peroxidation products in the NIDDM group. (D) Staining for PGC1α, showing diminished nuclear and cytoplasmic expression in the NIDDM group compared to the WT control group. Original magnification is consistent across panels. Scale bars = 100 μm. *** P < 0.001.

**Figure 8:**
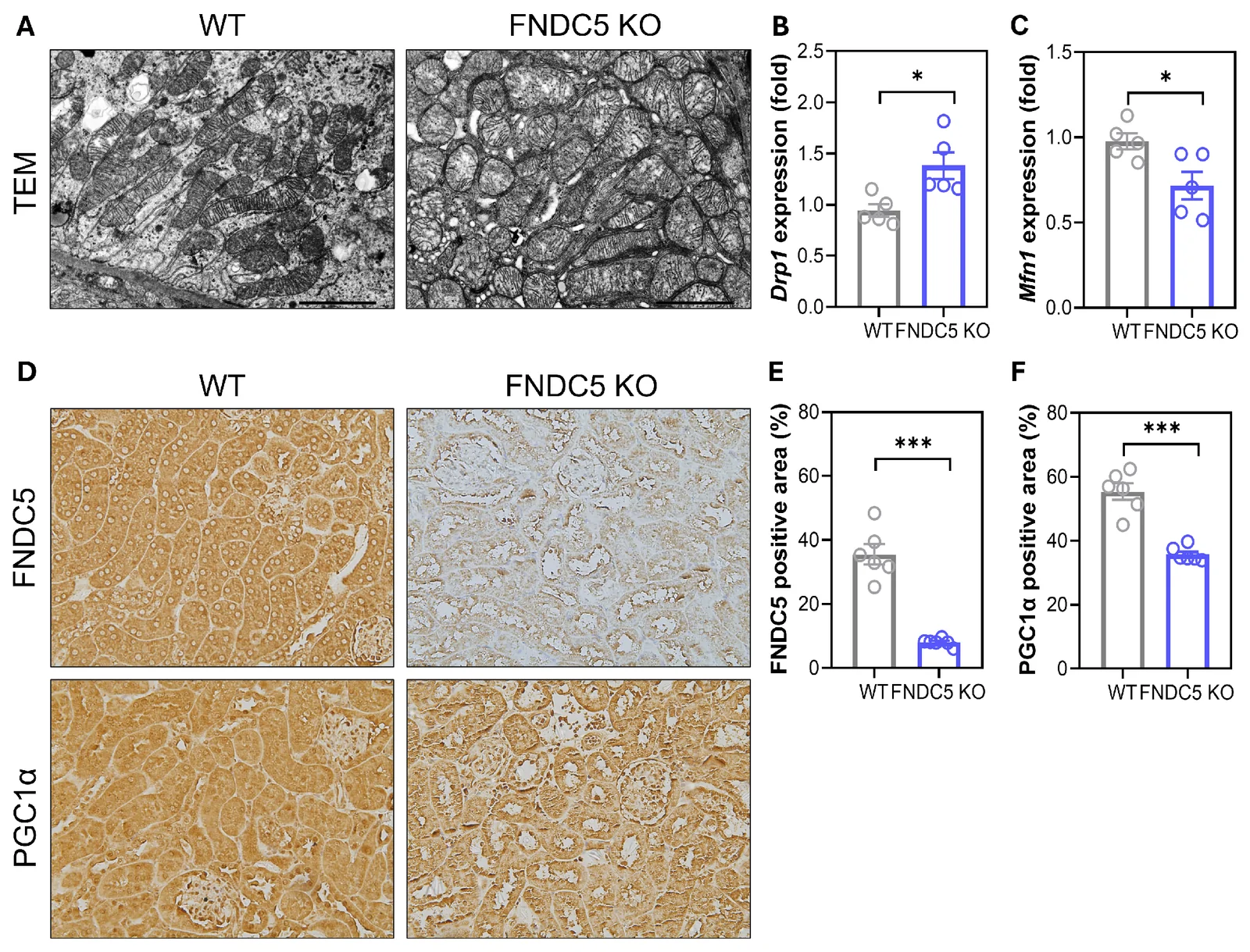
** Genetic deletion of *Fndc5* reproduces mitochondrial dynamics imbalance and reduced PGC1α expression under basal conditions.** (A) Representative transmission electron microscopy (TEM) images of kidney mitochondrial ultrastructure in WT and *Fndc5* KO mice (n = 6 mice/group). (B, C) Quantitative RT-PCR analysis of *Drp1* (B; n = 5 mice/group) and *Mfn1* (C; n = 5 mice/group) mRNA expression in kidney tissue from WT and *Fndc5* KO mice. (D) Representative immunohistochemical staining of FNDC5 and PGC1α in kidney sections from WT and *Fndc5* KO mice. (E, F) Quantification of FNDC5 (E) and PGC1α (F) positive staining area (%) in WT and *Fndc5* KO kidney sections (n = 6 mice/group). *P < 0.05, ***P < 0.001.

**Figure 9:**
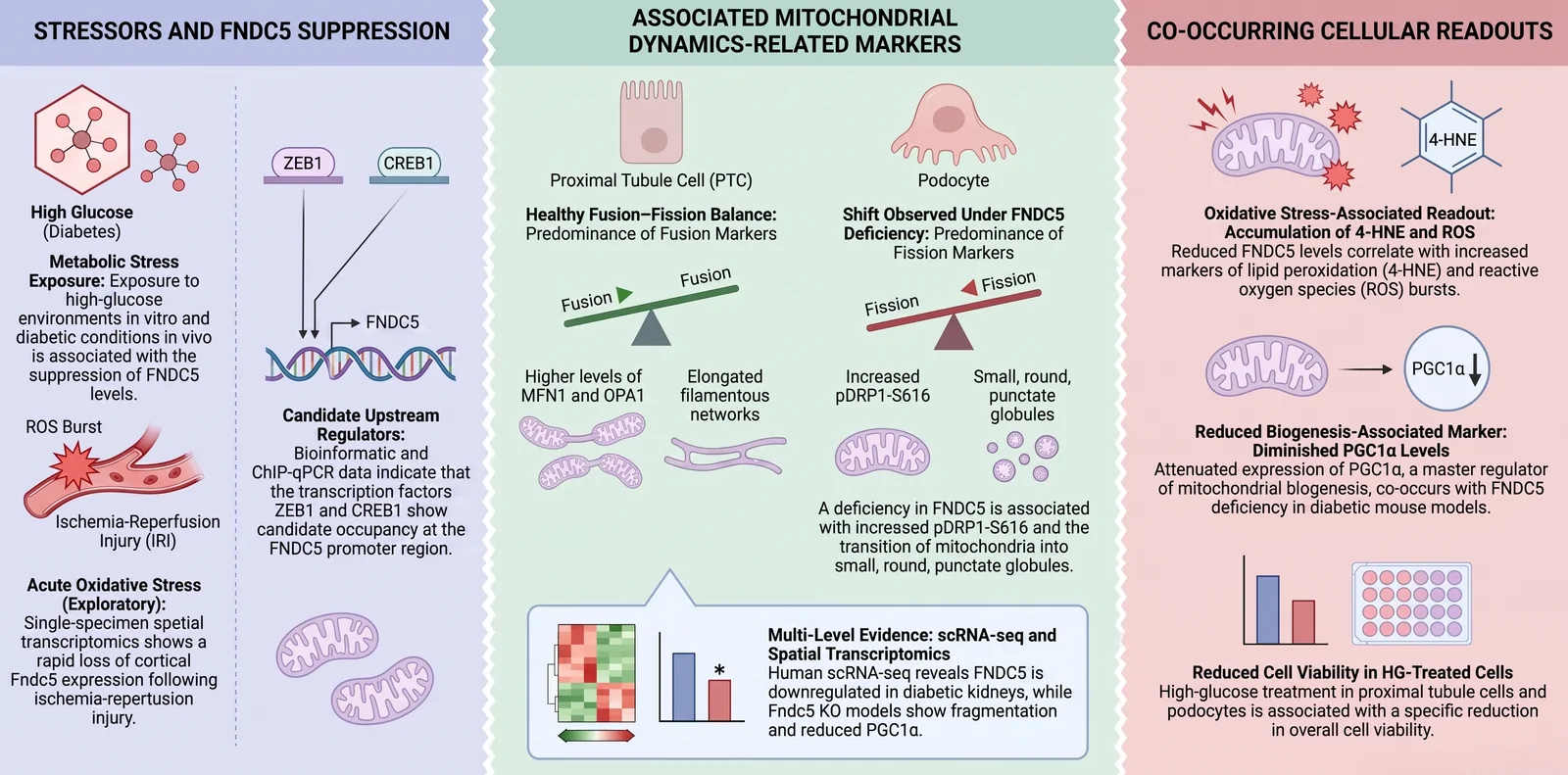
** FNDC5 and renal mitochondrial dynamics: an integrated association map.** This diagram summarizes the pattern of associations observed in this study and is not a validated mechanistic pathway. High glucose and ischemia-reperfusion injury (IRI; exploratory, single specimen) are both associated with reduced FNDC5 levels. ZEB1 and CREB1 show candidate occupancy at the FNDC5 promoter, requiring further validation with IgG and positive-control loci. FNDC5 deficiency co-occurs with a shift from fusion-predominant to fission-predominant markers and fragmented mitochondrial morphology in proximal tubule cells and podocytes, consistent with findings in human diabetic kidney scRNA-seq data and Fndc5 knockout mice. These changes co-occur with an oxidative stress-associated readout (4-HNE, ROS), reduced PGC1α expression, and reduced cell viability under high-glucose stress. Direct measures of mitochondrial function were not assessed, and the relationships shown have not been validated as a linear signaling cascade.

## Data Availability

The human kidney scRNA-seq dataset analyzed in this study is publicly available in the Gene Expression Omnibus (GEO) under accession number GSE131882. Other data supporting the findings of this study are available from the corresponding author upon reasonable request.
