# Supplementary material for: Association between FNDC5 Deficiency and Compromised Mitochondrial Integrity and Biogenesis in Kidney Disease
Source: Int J Med Sci. 2026 Sep 3;23(10):3281–95. doi: 10.7150/ijms.130226 (PMC13617500; doi:10.7150/ijms.130226)
Supplement: Supplementary file 1 [file ijmsv23p3281s1.pdf]

**Supplementary Material**

# Association between FNDC5 Deficiency and Compromised Mitochondrial Integrity and Biogenesis in Kidney Disease

Chien-Wei Huang, Chia-Jung Li, Junne-Ming Sung, Yau-Sheng Tsai*, Jin-Shuen Chen*

*These authors contributed equally to this work

**Supplementary Fig. S1. The full, uncropped, and unedited versions of the Western blots.**

**Supplementary Fig. S2. High glucose reduces cell viability in mouse podocytes.**

**Supplementary Fig. S3. Overexpression of FNDC5 modulates mitochondrial dynamics-related proteins under HG conditions.**

**Supplementary Fig. S4. Recombinant irisin attenuates high-glucose-induced oxidative stress in renal cells.**

**Supplementary Fig. S5. Metabolic and renal phenotypic characterization of the NIDDM mouse model.**

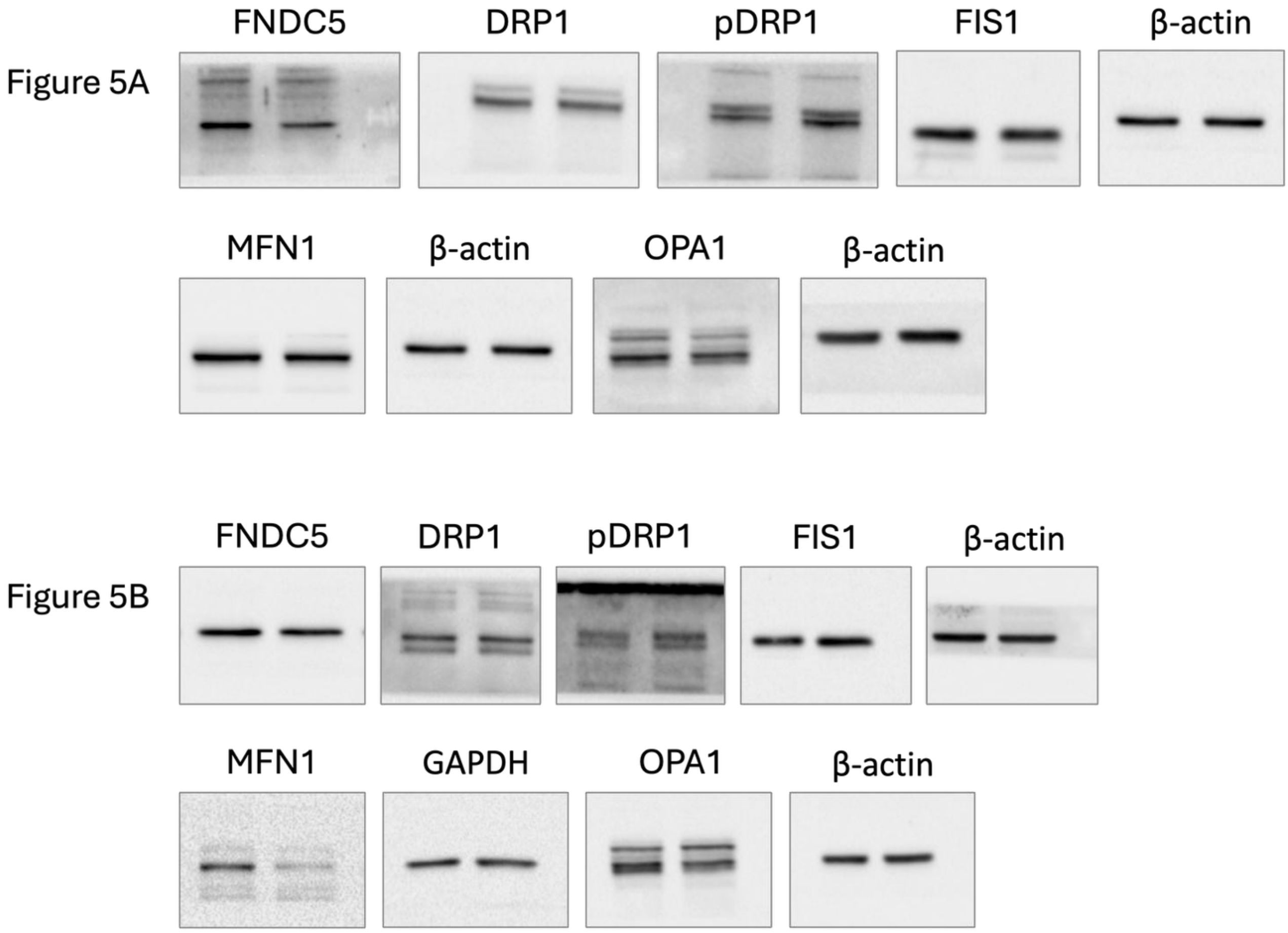


**Supplementary Fig. S1. The full, uncropped, and unedited versions of the Western blots.**

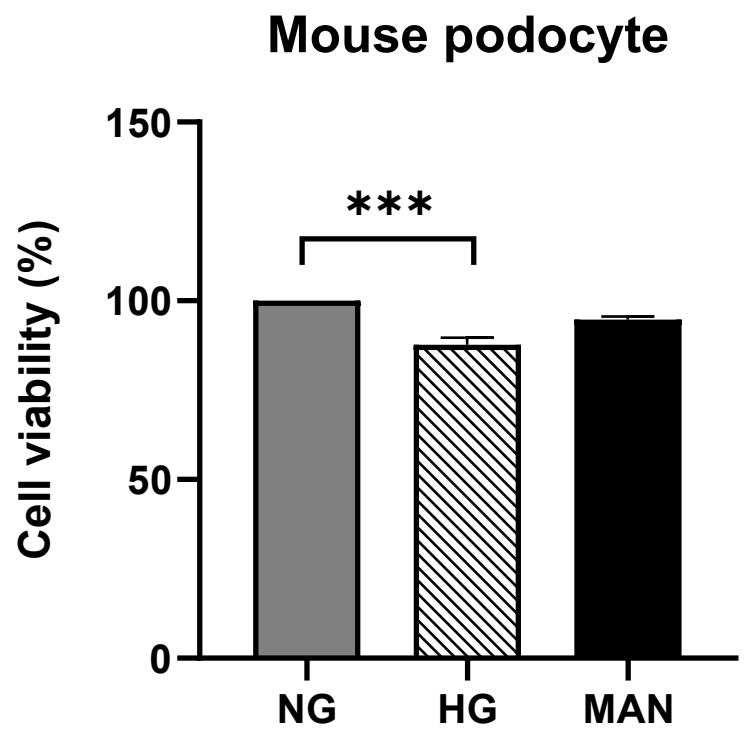


**Supplementary Fig. S2. High glucose reduces cell viability in mouse podocytes.** Cell viability of mouse podocytes was measured following treatment with NG (Normal Glucose, [5.5 mM]), HG (High Glucose, [33 mM]), or MAN (Mannitol, as an osmotic control). The results are expressed as a percentage relative to the NG group. Data are presented as mean ± SD/SEM. *** $P < 0.001$ indicates a significant difference between the NG and HG groups.

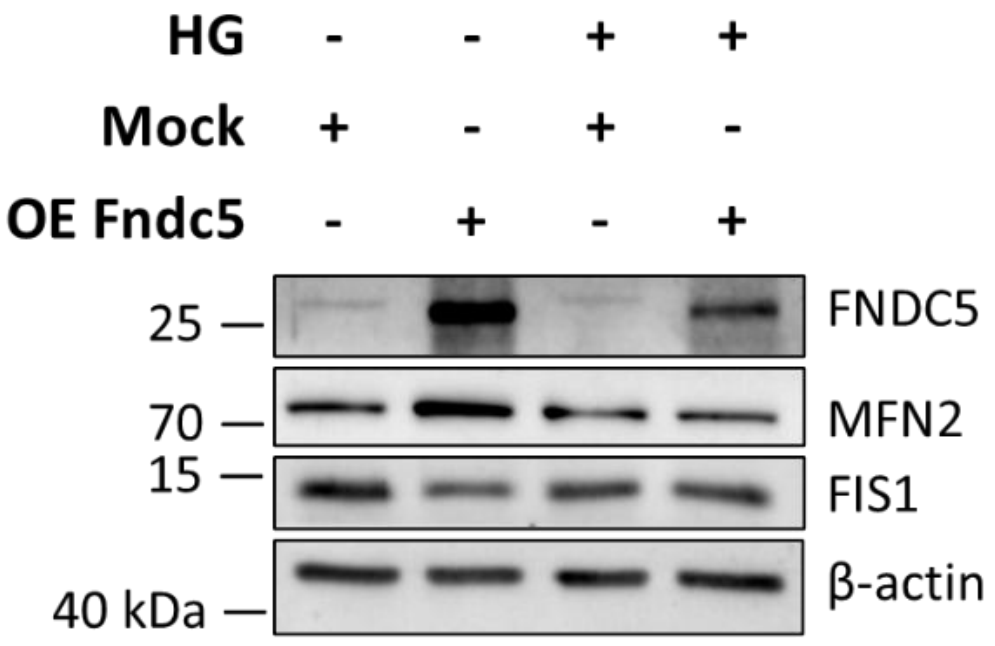


**Supplementary Fig. S3. Overexpression of FNDC5 modulates mitochondrial dynamics-related proteins under HG conditions.** Representative Western blot bands showing the protein expression levels of FNDC5, MFN2, and FIS1 in HK2 cells. Cells were treated with or without high glucose (HG) and transfected with either a mock vector or an FNDC5 overexpression plasmid. β-actin was used as an internal loading control to ensure equal protein loading across all lanes.

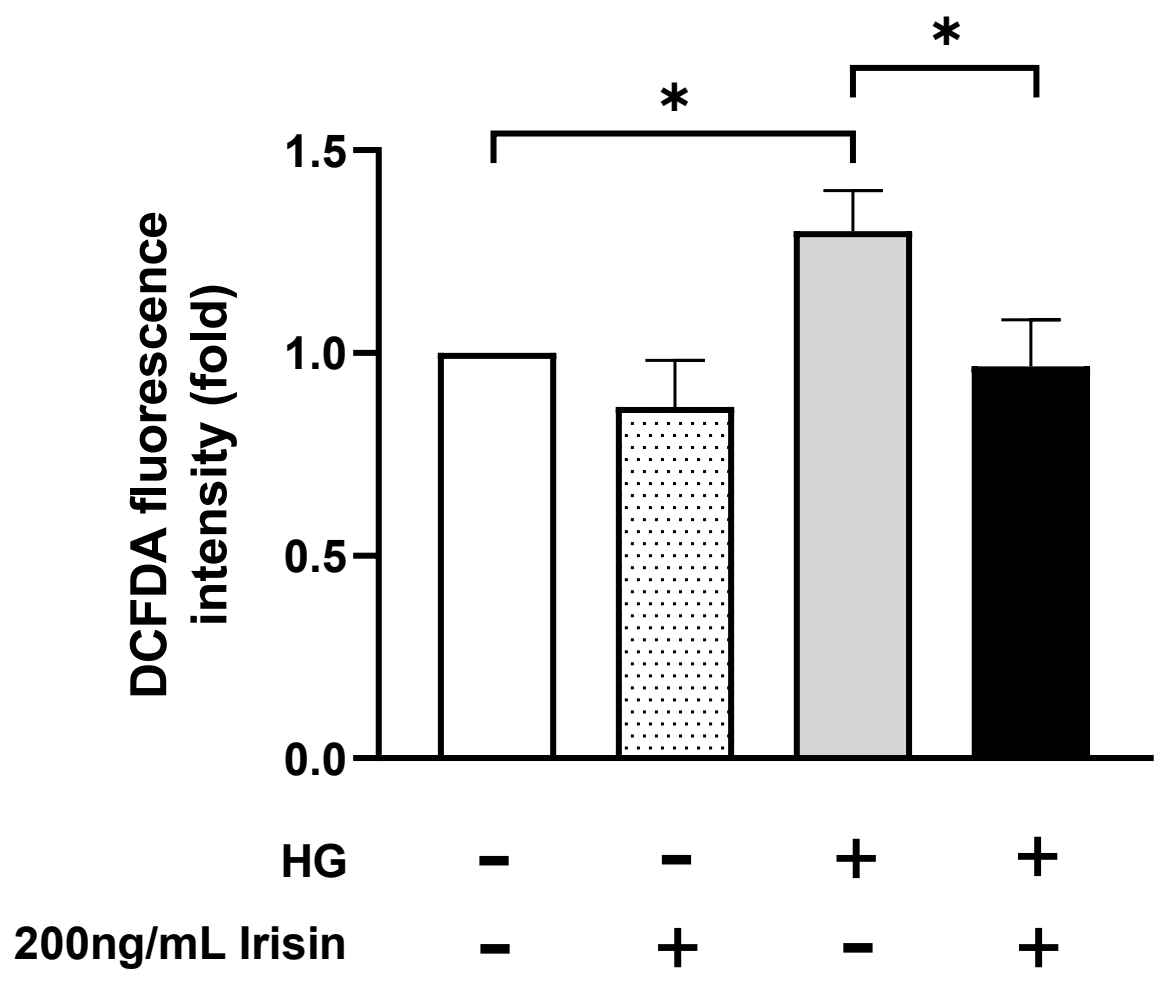


**Supplementary Fig. S4. Recombinant irisin attenuates high-glucose-induced oxidative stress in renal cells.** Intracellular ROS levels were measured using the DCFDA fluorescence assay. Human PTCs HK2 were treated with NG or HG in the presence or absence of recombinant Irisin (200 ng/mL) for 48 hours. The results are expressed as fold change in fluorescence intensity relative to the NG control group. Data are presented as mean ± SD (n =3). *P < 0.05 indicates statistical significance between the indicated groups.

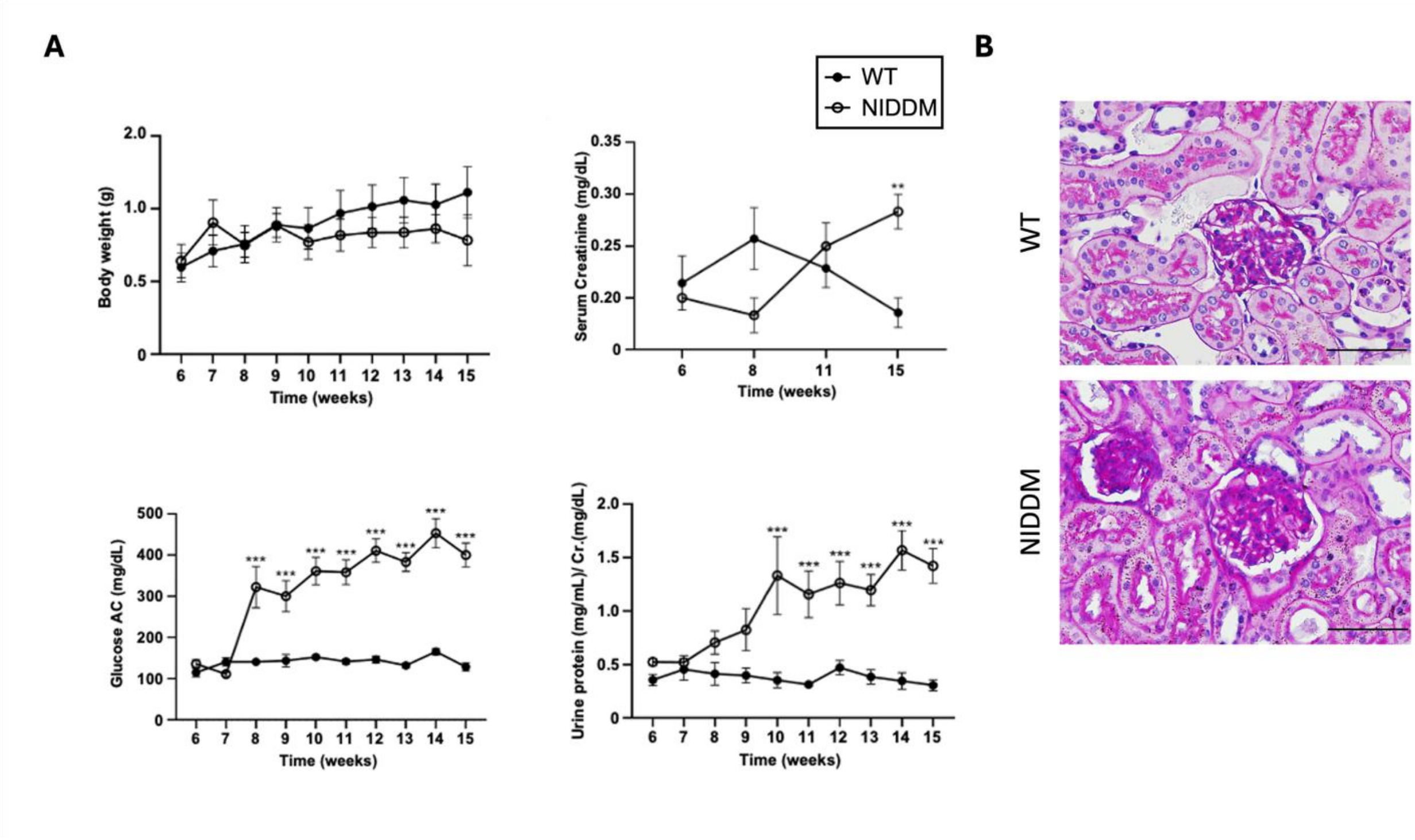


**Supplementary Fig. S5. Metabolic and renal phenotypic characterization of the NIDDM mouse model.** (A) Time course of body weight, fasting blood glucose levels, serum creatinine levels, and urinary protein-to-creatinine ratio in WT control and NIDDM mice. Data are presented as mean ± SEM (n = 6 for each group). (B) Glomerular basement membrane thickening and mesangial expansion were evaluated by periodic acid-Schiff staining in WT control and NIDDM mice. Scale bars = 100 μm.
